# Health Insurance Benefit Design and Healthcare Utilization in Northern Rural China

**DOI:** 10.1371/journal.pone.0050395

**Published:** 2012-11-21

**Authors:** Hong Wang, Yu Liu, Yan Zhu, Lei Xue, Martha Dale, Heather Sipsma, Elizabeth Bradley

**Affiliations:** 1 Department of Health Policy and Administration, School of Public Health, Yale University, New Haven, Connecticut, United States of America; 2 Bill & Melinda Gates Foundation, Seattle, Washington, United States of America; 3 Research Center for Healthcare Management, Tsinghua University, Beijing, China; University of Washington, United States of America

## Abstract

**Background:**

Poverty due to illness has become a substantial social problem in rural China since the collapse of the rural Cooperative Medical System in the early 1980s. Although the Chinese government introduced the New Rural Cooperative Medical Schemes (NRCMS) in 2003, the associations between different health insurance benefit package designs and healthcare utilization remain largely unknown. Accordingly, we sought to examine the impact of health insurance benefit design on health care utilization.

**Methods and Findings:**

We conducted a cross-sectional study using data from a household survey of 15,698 members of 4,209 randomly-selected households in 7 provinces, which were representative of the provinces along the north side of the Yellow River. Interviews were conducted face-to-face and in Mandarin. Our analytic sample included 9,762 respondents from 2,642 households. In each household, respondents indicated the type of health insurance benefit that the household had (coverage for inpatient care only or coverage for both inpatient and outpatient care) and the number of outpatient visits in the 30 days preceding the interview and the number of hospitalizations in the 365 days preceding the household interview. People who had both outpatient and inpatient coverage compared with inpatient coverage only had significantly more village-level outpatient visits, township-level outpatient visits, and total outpatient visits. Furthermore, the increased utilization of township and village-level outpatient care was experienced disproportionately by people who were poorer, whereas the increased inpatient utilization overall and at the county level was experienced disproportionately by people who were richer.

**Conclusion:**

The evidence from this study indicates that the design of health insurance benefits is an important policy tool that can affect the health services utilization and socioeconomic equity in service use at different levels. Without careful design, health insurance may not benefit those who are most in need of financial protection from health services expenses.

## Introduction

Following the abolishment of the cooperative economy during the economic reforms of the early 1980s, the rural Cooperative Medical System (CMS), a form of community-based health insurance that covered 90% of the communes, collapsed in rural China [1], [2]. The rural population covered by any form of health insurance dropped from 92.6% in 1976 to 6.1% in 1990 [3], and a 1998 China National Health Service Survey indicated that more than 87% of farmers still did not have any health insurance coverage and had to pay full medical expenses out-of-pocket [4]–[6]. After the CMS collapsed, access to basic health services became increasingly difficult for the majority of rural citizens, especially for the poor [7], [8]. In addition, large medical expenses have impoverished many rural families. Several studies have shown that 15 to 22% of poverty-stricken families in China became poor due to family members' health problems [9], [10]. Families often have to borrow money or sell their productive goods, such as seeds for the next season, in order to pay for medical expenses. Poverty due to illness has thus become a substantial social problem in rural China.

To provide financial protection against health care expenses, the Chinese government introduced the New Rural Cooperative Medical Schemes (NRCMS) in 2003 [5], [11]. Today, the NRCMS, which is a government sponsored voluntary health insurance program, covers more than 96% of the rural population in China [12] and operates at the county level with subsidized premiums from central, provincial, and local governments, in addition to a small proportion of individual contributions [13], [14]. Although reducing poverty due to illness was the principal goal of the NRCMS [11], [14], insurance benefit packages vary from one county to another, because local governments were free to choose a package and administrative arrangement that fit their local needs.

The NRCMS is an important step in terms of strengthening government responsibility in rural healthcare. Policymakers and researchers, however, are still debating how to design health insurance benefit packages, given resource constraints [15]. Because the primary goal is reducing poverty due to illness, the majority of insurance schemes focus on coverage for catastrophic illnesses with the NRCMS covering inpatient service only [12]. Nevertheless, this approach raises several concerns. The first one is whether health insurance covering prevention and early treatment delivered through outpatient services is more efficient and effective than catastrophic health insurance that focuses on hospitalization services [11]. The second one is whether the catastrophic health insurance that focuses on hospitalization services is more beneficial for people who are richer (i.e., is pro-rich) or for people who are poorer (i.e., pro-poor), given the fact that poor population faces more constraints than rich population to access hospitalization service [16].

Despite the ongoing debate about the impact of catastrophic health insurance in the NRCMS in rural China, the associations between different health insurance benefit package designs and healthcare utilization remain largely unknown. Existing studies have documented significant associations between NRCMS overall and increased healthcare utilization, particularly among high-income people [6], [17]–[24]. Nevertheless, we know little about how different health insurance benefit packages affect enrollees' utilization of outpatient and inpatient health services, and how these effects may differ by socio-economic status.

The debate about health insurance benefit designs is pertinent not only for the development of health insurance scheme in rural China but also for the development of universal health coverage (UHC), an increasingly important global initiative. UHC is defined as “securing access for all to appropriate preventive, curative, and rehabilitative services at an affordable cost” [25], [26]. Although UHC is meant to improve health care access for all people, early evidence from many developing countries displayed that many health insurance schemes developed in low-income countries only cover either outpatient or inpatient services due to resource constraints [27]. The most recent evidence from India suggests that UHC for catastrophic (hospital) care reduced the use of outpatient services and benefited the people who were non-poor more than people who were poor [28]. Our analysis of the China experience can contribute additional evidence about the impact of different health insurance benefit packages on overall utilization as well as the distributional results of insurance expansion efforts.

Accordingly, we sought to examine the impact of health insurance benefit design on health care utilization. We hypothesized that people with health insurance benefits that covered both outpatient and inpatient services compared with people with benefits that covered only inpatient services would be more likely to use outpatient services and less likely to use inpatient services. We also anticipated that health insurance benefits that covered both outpatient and inpatient services compared with health insurance benefits that covered only inpatient services would have a greater impact on health care utilization of people who were poor than people who were not poor.

## Methods

### Study Design and Sample

We conducted a cross-sectional study using data from a household survey of 15,698 rural residents of 4,209 households in 7 provinces (Shandong, Shanxi, Henan, Shannxi, Gansu, Ningxia, and Neimenggu), which were representative of the provinces along the north side of the Yellow River. The provinces are relatively underdeveloped in comparison with the region along Yanzi River, with a gradient from relatively richer areas downstream and relatively poorer areas upstream of Yellow River. Within each province, we selected 2 representative counties based on economic status (high and low income) and the acceptance by county governments; and then collaborated with the county official to identify 3 townships per county, including 1 high income, 1 medium income, and 1 low income township based on random sampling method and acceptance by township governments. Research teams visited 3 villages within each township, again including 1 high income, 1 medium income, and 1 low income village based on the random sampling method and traveling feasibility.

Households were randomly selected within each village with designed random sampling method. Each of the 14 research teams included 14 interviewers (undergraduates), 1 supervisor (MPH or PhD students) and 1 faculty member. Research teams approached the head of each household and explained the study. After obtaining informed consent, the researchers began by interviewing the head of household. If he or she were not available, the most senior person in the home was interviewed. All interviews were conducted face-to-face and in Mandarin. The survey was conducted on July 2010. For analysis, we excluded 5,936 respondents whose reported insurance coverage that was not plausible or not informative. This total included 1,788 respondents who reported having insurance that covered only outpatient care, which was not a benefit design that was offered in these provinces, 380 respondents who did not answer the insurance question, and 474 respondents who reported that they had no type of health insurance, and 3,294 respondents who did not know if they had health insurance, yielding an analytic sample of 9,762 respondents from 2,642 households.

### Measures

#### Health insurance benefit design

In each household, respondents indicated the type of health insurance benefit that the household had (coverage for inpatient care only or coverage for both inpatient and outpatient care). Although respondents' self-reported coverage status may differ from the actual coverage, we believe perceived coverage is likely most influential on utilization.

#### Health care utilization

Outpatient service utilization was measured by self-reported number of visits at different levels of health facilities (village clinic, township health center, and county hospital) in the 30 days preceding the household interview. Inpatient service utilization was measured by self-reported number of hospitalizations at different levels of health facilities (township health center and county hospital) in the 365 days preceding the household interview.

#### Demographic and socio-economic characteristics

We asked respondents to indicate their age, gender, education, employment status, number of people in the household, distance in kilometers from home to health facilities, and self-assessed health status (good, fair, or poor health). We also constructed a wealth index using principal component analysis (PCA) using data from the following housing characteristics and assets: type of house; type of toilet facility; drinking water source; access to internet; and ownership of a watch or clock, a bicycle, a refrigerator, a washer, a microwave oven, a radio, a black and white television, a color television, a VCD or DVD, a computer, a telephone, a cell phone, a motorcycle, a car or truck, and a tractor.

### Data analysis

We used standard descriptive statistics to describe the mean and standard deviation of respondent characteristics, health insurance benefit design, and health care utilization by level of care and by type of care. We examined the unadjusted association between health insurance benefit design and health care utilization (i.e., outpatient visits at the village, township, and county levels and hospitalization at the county and higher levels).

We used the classic count data model, negative binomial regression (NegBin), to estimate the association between health care utilization and health insurance benefit design because health care utilization is a nonnegative count variable with a large number of zeros [29]. We adjusted the models for individual demographic and socio-economic characteristics, as well as a set of county variable to capture the contextual differences at the county level. We also applied zero-inflated negative binomial model (ZINB) to account for excess zeros in the health care utilization variables. This model can be interpreted as a negative binomial regression included in a splitting mechanism that divides individuals into latent sub-populations of non-users of health services, with probability q, and potential-users, with probability 1-q [30]. We have applied these two models with separate estimations by facility level (village, township, and county levels) and type of health service (outpatient and inpatient) because the association between health insurance benefit package and utilization may differ by the facility level and by types of care.

We also compared the degree of socio-economic inequality in utilization between the two insurance types using the concentration index (CI) [31]. The CI is equal to 0 when there is perfect wealth equality. The CI is negative value when there is disproportionate concentration of health care utilization among people who are poorer. The CI is positive when there is a disproportionate concentration of health care utilization among people who are richer.

## Results

### Demographic and socio-economic characteristics of surveyed population

In our sample (N = 9,762), 52% of respondents had enrolled in health insurance that covered inpatient service only, and 48% of individuals enrolled in health insurance that covered both outpatient and inpatient services (**Table 1**). Slightly more than half (52%) of the sample were male; approximately 16% were under 15 years old, and24% were older than 55 years. Approximately 15% had received a high school level of education or higher. Half of the people in the sample were farmers, 10% were causal wage labor, and 10% were either business or regular wage employee. The average household size was 4.24 individuals, and the average distance to a village health clinic was 0.7 km. Approximately 64% of individuals assessed their health as good or excellent, 23% assessed it as fair, and 12% assessed it as poor.

**Table 1:** Characteristics of surveyed population (N = 9,762).

Variables	Definition	Mean	Standard Error
Insurance type			
instype0	covering inpatient care only	0.524	0.010
instype1	covering both inpatient & outpatient care	0.476	0.010
Demographic and SES characteristics			
Age			
agecat1	age< = 15	0.157	0.004
agecat2	age> = 16 and age < = 35	0.302	0.004
agecat3	age>35 and age< = 55	0.305	0.004
agecat4	age>55	0.236	0.005
Gender			
Female	female	0.476	0.004
Male	male	0.524	0.004
Education			
edu1	Below primary school	0.311	0.005
edu2	Primary school	0.207	0.004
edu3	Middle school	0.334	0.005
edu4	High school	0.115	0.004
edu5	College and above	0.033	0.002
Employment			
employ1	Business	0.052	0.003
employ2	Farmer	0.495	0.006
employ3	Regular wage employee	0.050	0.003
employ4	Casual wage labor	0.095	0.003
employ5	not working	0.155	0.004
Household size			
hsize	Number of people in the household	4.240	0.030
Distance to health facility			
distance	Distance from home to village clinic (km)	0.777	0.113
Wealth Index quintile			
Poorest	lowest 20%	−0.878	0.006
Second	20–40%	−0.596	0.003
Middle	40–60%	−0.371	0.003
Fourth	60–80%	0.043	0.010
Richest	highest 20%	1.806	0.030
Health status			
Self-assessed health status			
selfhlth1	good health	0.644	0.006
selfhlth2	fair health	0.233	0.006
selfhlth3	poor health	0.123	0.004

### Health care utilization

Among the sample population, 8.1% used outpatient services in the last 30 days; 7.6% used inpatient services in the last year. The average number of outpatient visits was 14.2 per 100 people during past 30 days. The average number of hospitalizations was 10.2 per 100 people during past year (**Table 2**).

**Table 2:** Unadjusted associations between health care utilization and health insurance benefit design.

Outcomes	Total		Insurance benefit design				
			Inpatient coverage only		Inpatient and outpatient coverage		P-value
	Encounter/person	Standard Error	Encounter/person	Standard Error	Encounter/person	Standard Error	
Utilization of outpatient services							
(No. outpatient visits in last month)							
Village clinics	0.092^1^	0.005	0.076	0.007	0.110	0.008	0.0005
Township health center	0.019	0.002	0.015	0.002	0.023	0.003	0.0156
County hospital	0.032	0.002	0.031	0.003	0.032	0.004	0.3868
Total outpatient services	0.142	0.006	0.122	0.008	0.165	0.010	0.0003
Utilization of inpatient services							
(No. hospitalization in past year)							
Township health center	0.022	0.002	0.024	0.003	0.019	0.003	0.8822
County hospital	0.081	0.004	0.078	0.005	0.084	0.005	0.2245
Total inpatient service	0.102	0.004	0.102	0.006	0.103	0.006	0.4639

1 0.092 can be interpreted as there were 9.2 village-level outpatien visits per 100 respondents in last month.

In unadjusted analysis, the design of the health insurance benefit was significantly associated with outpatient utilization, but not inpatient utilization. In particular, people who had both outpatient and inpatient care coverage compared with inpatient only coverage used village-level and township-level health facilities significantly more often and a great number of outpatient visits overall (P-values = 0.0005, 0.0156, and 0.0003) (**Table 2**).

We found similar results in the multivariable models, which were adjusted for gender, age, education, employment status, household size, wealth index, self-assessed health status, and distance from home to health facilities. People who had both outpatient and inpatient coverage compared with inpatient coverage only had significantly more village-level outpatient visits, township-level outpatient visits, and total outpatient visits (**Table 3**). Our results were robust across the two different approaches to multivariable modeling. The NegBin regression model indicated that people with outpatient and inpatient care coverage compared with people with only inpatient coverage had significantly higher village-level, township-level, and total outpatient utilization (incidence rate ratio (IRR = 1.343, P-value = 0.002; IRR = 1.361, P-value = 0.089; IRR = 1.244, P-value = 0.003, respectively). The ZINB model also indicated that the design of the health insurance benefit was associated with village-level, township-level, and total outpatient utilization (IRR = 1.501, P-value = 0.000, IRR = 1.464, P = value = 0.046, and IRR = 1.362, P-values = 0.000). Similar to the findings in the unadjusted analysis, the design of the health insurance benefit was not significantly associated with inpatient utilization.

**Table 3:** Adjusted associations between health insurance benefit design (comparing having both inpatient and outpatient coverage to having only inpatient coverage) and health care utilization.

Outcomes	NegBin Model			ZINB Model		
	Incidence Rate Ratio	Standard Error	P-value.	Incidence Rate Ratio	Standard Error	P-value
Utilization of outpatient services						
Village clinics	1.343	0.128	0.002	1.501	0.163	0.000
Township health center	1.361	0.246	0.089	1.464	0.280	0.046
County hospital	1.022	−0.143	0.877	0.989	0.151	0.941
Total outpatient services	1.244	0.093	0.003	1.362	0.116	0.000
Utilization of inpatient services						
Township health center	0.833	−0.137	0.266	0.798	0.137	0.190
County hospital	1.079	−0.090	0.364	1.075	0.094	0.412
Total inpatient service	1.021	−0.076	0.784	1.006	0.080	0.935

Note: Covariates in all models include gender, age, education level, employment status, household size, household wealth, self-assessed health, distance from home to health facilities, and county variables.

### The socio-economic inequality in healthcare utilization

We also found that the increased outpatient utilization associated with the health insurance benefit design favored people who were poorer compared with those who were wealthier. Among people who had coverage for both outpatient and inpatient services, the CIs for total outpatient services were negative, indicating that poorer respondents were more likely than the wealthier respondents to use outpatient services (CI = −0.062, P-value<0.10) (**Table 4**); however, this pro-poor utilization pattern was only apparent for total outpatient services and outpatient services at the village and township levels (CI = −0.062, −0.101 and −0.127, respectively, P-values<0.10). At the county level, in contrast, the CI was positive (CI = +0.116, P-value<0.10), indicating that wealthier respondents were more likely than poorer respondents to use county-level outpatient services at this level. Among people who had health insurance benefit designs that only covered inpatient care, the CIs were not significantly different from zero.

**Table 4:** Distribution in outpatient service utilization by health insurance benefit design.

Wealth index (Quintile)	Total		Village clinic		Township health center		County hospital	
	Inpatient only	Inpatient & outpatient	Inpatient only	Inpatient & outpatient	Inpatient only	Inpatient & outpatient	Inpatient only	Inpatient & outpatient
Poorest	0.139	0.215	0.093	0.159	0.015	0.026	0.031	0.031
2nd	0.139	0.166	0.086	0.105	0.020	0.033	0.033	0.028
Middle	0.122	0.191	0.084	0.122	0.013	0.032	0.025	0.037
4th	0.105	0.125	0.063	0.088	0.012	0.013	0.031	0.024
Richest	0.105	0.126	0.055	0.074	0.017	0.010	0.033	0.042
CI	−0.017	**−0.062**	−0.055	**−0.101**	0.063	**−0.127**	0.040	**0.116**
se(CI)	0.034	0.031	0.045	0.039	0.082	0.067	0.060	0.066
t-test(CI)	−0.49	−1.98	−1.24	−2.59	0.77	−1.90	0.67	1.78

Note: Bold CI indicate significant difference from zero at 10%.

For total inpatient utilization and county-level inpatient utilization, the CIs among people with insurance covering both inpatient and outpatient service were positive (CI = 0.061, CI = 0.062, respectively, P-values<0.10, respectively) (**Table 5**), indicating that wealthier respondents were more likely than poorer respondents to use inpatient services overall and at the county hospitals. The CIs among people with insurance covering inpatient services only were not significantly different from zero for county-level, township-level, or total inpatient utilization.

**Table 5:** Distribution in inpatient service utilization by health insurance benefit design.

Wealth index (Quintile)	Total		Township health center		County hospital	
	Inpatient only	Inpatient & outpatient	Inpatient only	Inpatient & outpatient	Inpatient only	Inpatient & outpatient
Poorest	0.118	0.100	0.024	0.017	0.094	0.083
2nd	0.106	0.088	0.042	0.017	0.064	0.071
Middle	0.100	0.096	0.013	0.024	0.087	0.072
4th	0.090	0.142	0.021	0.024	0.068	0.118
Richest	0.097	0.094	0.019	0.014	0.079	0.080
CI	−0.009	**0.061**	−0.102	0.056	0.019	**0.062**
se(CI)	0.035	0.032	0.078	0.072	0.039	0.037
t-test(CI)	−0.26	1.87	−1.31	0.78	0.50	1.68

Note: Bold CI indicate significant difference from zero at 10%.

## Discussion

We found that having the more comprehensive health insurance benefit design, which covered both outpatient and inpatient services rather than inpatient services only, was associated with significantly greater utilization of village-level, township-level, and total outpatient services. This is consistent with the notion that insurance would provide economic relief from out-of-pocket payments for outpatient care; nevertheless, we did not find significant difference in outpatient services at the county level, perhaps because such care would require time away from work and high transportation costs to access. Insurance coverage for the services may not have been enough to defray other expenses incurred in order to increase the access of county-level services. Previous studies have shown that time and travel costs can be a substantial impediment to accessing services, especially in rural and low-income settings [32]. The lack of increase in the county-level outpatient services in the context of increased health insurance benefits indicates a relatively price inelastic demand for county-level outpatient care.

We found mixed results for whether the benefits of increased access to outpatient services conferred by more comprehensive health insurance were progressive (pro-poor) or regressive (pro-rich). The increases in village-level, township-level, and total outpatient utilization are disproportionately experienced by people with lower income levels at 90% confidence level. At the same time, the outpatient services at the county hospital level are disproportionately used by people at higher income levels. These findings are consistent with our expectations. People with lower incomes were more sensitive to the price of health services than people with higher incomes. With health insurance coverage for outpatient services at the village and township levels, increases in outpatient service utilization at these two levels was more apparent among people with lower incomes. Therefore, the policy effect at these two levels was pro-poor. In contrast, the increased use of the county-level outpatient services among the people with higher incomes suggests that the policy effect at the county-level was more pro-rich than pro-poor.

In contrast, inpatient services at the county hospital level were disproportionately utilized by people at higher income levels. It is possible that increased utilization of outpatient services among people with lower incomes may attenuate their demand for inpatient services. Similarly, as transportation costs and other lost work opportunity costs may be higher for inpatient services overall and at the county level, such services may be more feasible for people with high incomes. Our findings suggest that health insurance that only covers inpatient services may result in reductions in outpatient utilization, particularly at the village and township levels and among people with lower income. This result is consistent with concerns voiced by the World Health Organization (WHO 2010) that people who are provided insurance only for inpatient service may delay critical outpatient and preventive services that can protect their longer-term health and reduce catastrophic illness. Chinese government at various levels are the major contributors to the NRCMS premiums; these investments are more likely to benefit to the poor if they are used to provide insurance that covers both outpatient and inpatient services rather than just inpatient services.

Many developing countries have adopted supply-side policies, which direct investment to health care facilities directly, in order to increase health service accessibility; nevertheless, recent evidence indicates that such policies benefit middle- and higher-income people disproportionately [33]–[35]. Our findings suggest that if the Chinese government through NRCMS can provide health insurance that covers both outpatient and inpatient services, such policies may be pro-poor rather pro-rich for both village and township-level outpatient care. Therefore, the appropriate health insurance benefit package design could improve equity in distribution of the government investment in healthcare delivery system.

Our results should be interpreted in light of some limitations. First, we ascertained health insurance benefit designs using self-reported data, which may have inaccuracies; nevertheless, because utilization choices are generally made based on perceived coverage, we believe the self-reported data are appropriate for this study. Second, although our sample was large, we were restricted to a pre-specified region of China, and results in other parts of China may differ. Last, we were unable to measure the impact of various health insurance benefit designs on health status, which may be interesting in evaluating NRCMS. Previous studies have examined this issue and reported no association between NRCMS and self-reported health status or sickness or injury in the past four weeks [36], or between NRCMS and mortality rates among pregnant women or young children at the village level [22]. We focused our inquiry on increasing service utilization, which from the perspective of financial protection for the population, remains an important health policy objective for the Chinese government.

## Conclusion

The evidence from this study indicates that the design of health insurance benefits is an important policy tool that can affect the health services utilization and socioeconomic equity in service use at different levels. Without careful design, health insurance may not benefit those who are most in need of financial protection from health services expenses.
